# Einsatz von Biosimilars in der Behandlung der rheumatoiden Arthritis: Ein Überblick

**DOI:** 10.1007/s00393-021-01129-6

**Published:** 2021-11-26

**Authors:** Harriet Morf, Thorben Witte

**Affiliations:** 1 Medizinische Klinik 3 – Rheumatologie und Immunologie, Friedrich-Alexander Universität Erlangen-Nürnberg und Universitätsklinikum Erlangen, Ulmenweg 18, 91054 Erlangen, Deutschland; 2 Klinik für Rheumatologie und klinische Immunologie, Johannes Wesling Klinikum Minden, Minden, Deutschland

**Keywords:** Referenzarzneimittel, Kosten, Austauschbarkeit, Sicherheit, Nebenwirkungen, Reference drug, Costs, Interchangeability, Safety, Side effects

## Abstract

In der Therapie der rheumatoiden Arthritis sind seit ca. 20 Jahren Biologika ein fester Bestandteil. Da Arzneimittelpatente in der Regel nach 10 Jahren auslaufen, wurden in den letzten Jahren Biosimilars auf den Markt gebracht. In vielen Studien konnte gezeigt werden, dass sie bei vergleichbarer Sicherheit und Effektivität eine gleichwertige Alternative zum Referenzarzneimittel darstellen. In manchen Fällen zeigten sich sogar geringere Raten an unerwünschten Arzneimittelwirkungen im Vergleich zu den Referenzarzneimitteln. Weiterhin können durch Biosimilars erhebliche Kosten eingespart werden, die sich allein in Deutschland auf einen jährlichen dreistelligen Millionenbetrag belaufen. Dabei lassen sich große regionale Unterschiede bei der Verschreibungshäufigkeit von Biosimilars in Deutschland ausmachen, die sich auch im Einsparungspotenzial widerspiegeln. Eine Umstellung auf ein Biosimilar ist unter Einbezug des Patienten gut möglich und auch erwünscht. In diesem Sinne ist auch die Erstverschreibung eines Biosimilars statt des Referenzarzneimittels zu befürworten.

## Abstract (en)

Biologics have been an integral part of the treatment of rheumatoid arthritis for approximately 20 years. As patents for pharmaceuticals generally expire after 10 years, in recent years biosimilars have come onto the market. Many studies have shown that they are an equivalent alternative to the reference drug with comparable safety and efficacy. In some cases, they even showed lower rates of adverse drug reactions compared to the reference drugs. Furthermore, considerable costs can be saved by biosimilars, which amount to an annual three-digit million sum in Germany alone. Large regional differences in the prescription frequency of biosimilars in Germany can be identified, which are also reflected in the savings potential. A switch to a biosimilar is possible and desirable with the involvement of the patient. In this sense, the initial prescription of a biosimilar instead of the reference drug is also to be advocated.

In der Therapie der rheumatoiden Arthritis finden zahlreiche Arzneimittel Verwendung. Neben klassischen Therapeutika wie Prednisolon und Methotrexat haben in den letzten 2 Jahrzehnten die Biologika einen breiten Einzug in die Therapie autoimmunologischer Erkrankungen erhalten (Erstzulassung Infliximab 1999) [1] und die Therapieoptionen damit wesentlich vergrößert. Mit Ablauf des Patentschutzes dieser Biologika ergänzen Biosimilars das Arzneimittelangebot. Dieser Wandel begann vor ca. 8 Jahren in der Therapie der rheumatoiden Arthritis mit den Biosimilars zu Infliximab [2]. Aktuell gibt es für die 3 TNF(Tumornekrosefaktor)-α-Antagonisten (Etanercept, Adalimumab, Infliximab) sowie für den B‑Zell-Antagonisten Rituximab Biosimilarvarianten. Im Folgenden sollen der Nutzen sowie der Einsatz dieser Biosimilars im Hinblick auf die rheumatoide Arthritis näher beleuchtet werden.

## Definition

Laut der Europäischen Arzneimittelbehörde (EMA) sind Biologika Medikamente, die aktive Wirkstoffe enthalten und aus biologischer Quelle stammen [3]. Diese Quellen können lebende Zellen oder Organismen sein. In der Mehrzahl der Fälle basieren Biologika auf Eiweißstrukturen, die von einfachen Proteinen bis hin zu komplexen Antikörpern reichen [3].

Aufgrund des auslaufenden Patentschutzes (10 Jahre nach Zulassung) für einige Biologika kamen die sog. Biosimilars auf den Markt. Biosimilars sind Biologika, die ihrem Referenzarzneimittel (Originalpräparat) sehr ähnlich sind. Einziger Unterschied zu dem Referenzarzneimittel ist der Herstellungsprozess. Durch strenge Kontrollen wird sichergestellt, dass Wirkungsweise und Sicherheit sich nur geringfügig unterscheiden und somit keine klinische Relevanz haben [3]. Laut EMA müssen Biosimilars zu ihrem Referenzarzneimittel folgende spezifische Eigenschaften erfüllen:

- hohe Ähnlichkeit,
- keine bedeutsamen klinischen Unterschiede,
- Variabilität innerhalb strenger Grenzen,
- gleiche Standards bezüglich Sicherheit, Qualität und Wirksamkeit.

Zu differenzieren sind Biosimilars von Generika. Während Generika eine exakte Nachbildung der Referenzarzneimittel sind, sind Biosimilars durch eine natürliche Variabilität sowie ihren komplexen Herstellungsprozess keine exakte Nachbildung ihres Referenzarzneimittels. Dies beruht darauf, dass biologische Arzneimittel von Organismen produziert werden, die naturbedingt eine gewisse Variabilität aufweisen. So können die biologische Aktivität und die Aminosäuresequenz gleich sein, die Glykosylierung kann sich jedoch in verschiedenen Chargen unterscheiden. Daher müssen Biosimilars vor ihrer Zulassung zusätzliche Untersuchungen wie Vergleichbarkeitsstudien mit dem Referenzarzneimittel durchlaufen, bis sie zugelassen werden können. Diese Vergleichbarkeitsstudien laufen in 3 Schritten ab: Zu Beginn werden vergleichende Qualitätsstudien durchgeführt, danach vergleichende nichtklinische Studien und anschließend vergleichende klinische Studien [3].

## Zugelassene Biosimilars

Bis heute sind einige Biosimilars für die Therapie der rheumatoiden Arthritis zugelassen. Die Tab. 1 gibt hierzu einen Überblick.

**Table 1:** 

Wirkstoff	Biosimilar	Zulassung durch EMA	Hersteller
*Adalimumab*	Amgevita™	03/2017	Amgen®
	Solymbic®	03/2017	Amgen®
	Imraldi™	08/2017	Biogen™
	Hulio™	09/2018	Mylan®
	Hyrimoz®	07/2018	Sandoz®
	Hefiya®	07/2018	Sandoz®
	Idacio®	04/2019	Fresenius Kabi
	Amsparity™	02/2020	Pfizer
	Yuflyma®	02/2021	Celltrion™
*Etanercept*	Benepali®	01/2016	Samsung Bioepis
	Erelzi®	06/2017	Sandoz®
	Nepexto®	06/2020	Mylan®
*Infliximab*	Inflectra®	09/2013	Pfizer
	Remsima®	09/2013	Celltrion™
	Flixabi®	05/2016	Samsung Bioepis
	Zessly®	05/2018	Sandoz®
*Rituximab*	Truxima®	02/2017	Celltrion™
	Rixathon®	05/2017	Sandoz®
	Riximyo®	05/2017	Sandoz®
	Ritemvia®	07/2017	Celltrion™
	Ruxience®	04/2020	Pfizer

*EMA* Europäische Arzneimittelbehörde

## Sicherheit und Nebenwirkungen

Neben der Prüfung der EMA bezüglich Sicherheit und Wirksamkeit der Biosimilars im Vergleich zu den Referenzpräparaten, wurden Studien zur Vergleichbarkeit einzelner Biosimilars untereinander durchgeführt.

Für *Adalimumab* wurde eine Studie zwischen Imraldi™ (Biogen™, Cambridge, MA, USA) und dem Referenzarzneimittel (Humira®, AbbVie, Lake Bluff, IL, USA) durchgeführt. Geprüft wurde hier in einer randomisierten, doppelblinden Studie mit 544 Patienten (269 Biosimilar, 273 Referenzarzneimittel), die mit Methotrexat vorbehandelt wurden, aber alle Biologika-naiv waren. Der primäre Endpunkt war das ACR20(American College of Rheumatology 20)-Ansprechen nach 24 Wochen. Hier erreichten nach 24 Wochen 72,4 % der Patienten im Biosimilararm und 72,2 % der Patienten im Referenzarzneimittelarm den primären Endpunkt. Zeitgleich wurden die unerwünschten Arzneimittelwirkungen (UAWs) beobachtet. Dort zeigte sich, dass innerhalb der ersten 24 Wochen 0,7 % in der Biosimilar und 3,3 % in der Referenzarzneimittelgruppe einen Therapieabbruch aufgrund der UAWs durchführten. Schwerwiegende Infektionen traten bei < 1 % der Patienten in den jeweiligen Gruppen auf, keine davon war eine aktive Tuberkulose [5]. In der Imraldi™-Gruppe zeigte sich zudem eine geringere Rate an Nebenwirkungen an der Injektionsstelle (32 Reaktionen bei 4 Patienten unter Imraldi™, 9 Reaktionen bei 8 Patienten in der Referenzarzneimittelgruppe). Dies führte insgesamt zu einer geringeren Therapieabbruchrate in der Imraldi™-Gruppe. Für beide Gruppen lässt sich zudem festhalten, dass es vergleichbare Fälle von Antikörperentwicklung gegen die Biologika gab [6].

Der Vergleich des Biosimilars Adalimumab (Cyltezo™, Boehringer Ingelheim, Ingelheim am Rhein, Deutschland) zum Referenzarzneimittel ergab ähnliche Ansprechraten. Die Studie von Cohen et al. war eine randomisierte, doppelblinde, prospektive Studie mit RA(rheumatoide Arthritis)-Patienten unter Methotrexat als Therapeutikum. Primärer Endpunkt war eine ACR20-Ansprechrate zum Zeitpunkt 12 und 24 Wochen. Wirkung und Sicherheit wurden im Follow-up bis zu 52 Wochen lang beobachtet. Insgesamt wurden 645 Patienten eingeschlossen, davon wurden 324 Patienten auf das Biosimilar eingestellt und 321 auf das Referenzarzneimittel. Nach 24 Wochen erreichten 69 % der Biosimilarpatienten und 64,5 % der Referenzarzneimittelgruppe ein ACR20-Ansprechen. Daraufhin wurden 3 neue Studienarme mit insgesamt 593 Patienten eröffnet. Jeweils ein Arm behielt die Therapie mit dem Referenzarzneimittel, der andere Arm blieb bei dem Biosimilar, und der dritte Arm führte einen Wechsel von Referenzarzneimittel zu Biosimilar durch. Über diesen Zeitraum waren die ACR20/ACR50/ACR70-Raten vergleichbar in allen 3 Gruppen. Die Sicherheit bzw. die UAWs der Arzneimittel waren in allen Therapiearmen über den gesamten Zeitraum vergleichbar. UAWs waren Pyelonephritis, Urtikaria, Appendizitis, septische Arthritis und Bronchitis. Auch eine Antikörperbildung gegen die Biologika war in den Vergleichsgruppen identisch [7].

In einer Arbeit zu *Infliximab* konnten ähnliche Daten erhoben werden. Kameda et al. führten eine doppelblinde, randomisierte, multizentrische Studie an 174 Zentren in 28 Ländern durch. Dabei wurde das Referenzarzneimittel Infliximab mit Inflectra® (Pfizer, New York City, NY, USA) verglichen. Eingeschlossen wurden nur Infliximab-naive Patienten bzw. Patienten ohne Lymphozyten-depletierende Therapie: 324 Patienten in der Biosimilargruppe und 326 Patienten in der Referenzarzneimittelgruppe. Die Studienpopulation verhielt sich wie folgt: Etwa 80 % der Patienten waren Frauen. Regionen, in denen die Studie durchgeführt wurde, waren u. a. Japan, Westeuropa, Lateinamerika und Nordamerika.

Alle Studienteilnehmer erhielten Methotrexat (MTX) und wurden zu Beginn der Studie mit dem Biosimilar oder dem Referenzarzneimittel behandelt. Nach 30 Wochen wurden 50 % der Referenzarzneimittelgruppe auf das Biosimilar umgestellt, in Woche 54 wurden schließlich auch die verbleibenden Teilnehmer der Referenzarzneimittelgruppe auf das Biosimilar umgestellt. Primärer Endpunkt war ein ACR20-Ansprechen in der Woche 14, sekundäre Endpunkte waren ein ACR20/DAS28(Disease Activity Score 28)-CRP(C-reaktives Protein)-Ansprechen in der 30. Woche. Gezeigt werden konnte, dass unabhängig von Alter, Ethnie, Geschlecht oder Wohnort ein ACR20-Ansprechen zum Zeitpunkt der 14. Woche in beiden Gruppen vergleichbar war. Dies konnte auch für den Zeitpunkt der 30. Woche nachgewiesen werden [8]. Somit ist auch hier eine gleiche Effektivität zwischen Biosimilar und Referenzarzneimittel gezeigt worden.

Bezüglich Sicherheit führten Kim et al. eine 5‑jährige, retrospektive Studie zu Infliximab bei Patienten mit rheumatoider Arthritis (154 Patienten) und ankylosierender Spondylitis (337 Patienten) durch. Dabei waren die Patienten Infliximab-naiv oder wechselten vom Referenzarzneimittel zum Biosimilar Remsima® (Celltrion™, Incheon, Südkorea). Unter den Patienten mit rheumatoider Arthritis entwickelten 31,8 % UAWs. Am häufigsten wurden respiratorische Infekte beobachtet, darüber hinaus traten auch grippeähnliche Symptome oder Urtikaria auf. Hier war kein signifikanter Unterschied zwischen den beiden Gruppen messbar. Malignome waren nicht nachweisbar. In der gesamten Studie wurden 22 schwerwiegende UAWs beobachtet, 19 davon bei Biologika-naiven Patienten. Lediglich 5 Patienten der gesamten Studie mussten aufgrund schwerer UAWs die Therapie beenden (Tuberkulose, Milzabszess, Hypersensitivitätsvaskulitis, Serumkrankheit). Laut Studie wurden 3 Tuberkulosefälle (2 Reaktivierungen und 1 Neuerkrankung) beobachtet, wobei einer davon bei einem Patienten mit einer rheumatoiden Arthritis auftrat. Die Serumkrankheit und die Hypersensitivitätsvaskulitis traten bei Patienten mit ankylosierender Spondylitis auf. Über den Patienten mit Milzabszess wurden keine weiteren Angaben gemacht [9]. Somit zeigt auch diese Studie eine hohe Sicherheit des Biosimilars.

Auch für das *Etanercept* wurden Vergleichsstudien mit Biosimilars durchgeführt, die gleiche Effektivität und Sicherheit von Letzterem zeigten. Emery et al. führten eine doppelblinde, randomisierte Studie mit 245 Patienten zu Etanercept durch. Alle Patienten erhielten parallel zur Studienmedikation MTX. Zum Startzeitpunkt wurde das Patientenkollektiv in 2 Arme aufgeteilt, ein Arm erhielt das Referenzarzneimittel und der andere Arm das Biosimilar Benepali® (Samsung Bioepis, Incheon, Südkorea). In der 52. Woche wurde der Arm mit dem Referenzarzneimittel auf das Biosimilar umgestellt. Der komplette Beobachtungszeitraum ging bis zur Woche 100. Es konnte gezeigt werden, dass in der 52. Woche vergleichbare ACR20/50/70-Ansprechraten in beiden Armen auftraten. Auch nach dem Wechsel zeigten sich ähnliche Ansprechraten in der 100. Woche; 47,6 % (*n* = 60) der Patienten, die das Biosimilar durchgängig erhielten, entwickelte UAWs; 48,7 % (*n* = 58) der Patienten, die auf das Biosimilar wechselten. Am häufigsten zeigten sich respiratorische Infekte (7,9 % mit durchgehend Biosimilar und 7,6 % im wechselnden Arm). Vier Patienten in der wechselnden Gruppe bzw. 2 Patienten in der anderen Gruppe entwickelten UAWs, die zu einer Beendigung der Therapie mit Etanercept führten. Nähere Informationen waren dazu nicht einsehbar. Darüber hinaus entwickelte jeweils 1 Patient pro Therapiearm Antikörper gegen das Biologikum, woraus sich aber keine Neutralisierung der Therapeutika entwickelte [10].

Zuletzt zeigten auch Biosimilars von Rituximab in mehreren Vergleichsstudien ein ähnliches Verhalten bezüglich Effektivität und Sicherheit gegenüber dem Referenzarzneimittel. In einer Metaanalyse von 6 randomisierten, kontrollierten Studien mit insgesamt 1744 Patienten zeigte sich im ACR20/50/70-Ansprechen nach 24 bzw. 48 Wochen kein signifikanter Unterschied zwischen der Biosimilargruppe und der Referenzarzneimittelgruppe. Darüber hinaus waren die Raten an Antikörperentwicklungen gegen das Arzneimittel in beiden Gruppen vergleichbar. Nähere Angaben zu UAWs wurden in dieser Analyse nicht gemacht [11].

Nach aktueller Datenlage sind Biosimilars sicher und effektiv

Zusammenfassend lässt sich festhalten, dass die aktuelle Datenlage nahelegt, dass Biosimilars sicher und effektiv sind. Es bleibt eine jeweilige Einzelfallentscheidung mit dem Patienten, ob das Referenzarzneimittel oder ein Biosimilar gewählt wird. Ausschlaggebend sollte dabei das jeweilige Beratungsgespräch des Rheumatologen mit dem Patienten sein.

Allgemein senken Biosimilars die Therapiekosten bei ähnlicher Effektivität. Weiterhin legen die vorliegenden Daten nahe, dass für einige Präparate sogar die unerwünschten Arzneimittelwirkungen niedriger sein können.

## Austauschbarkeit von Biosimilars

Generell dürfen Biosimilars nicht ohne ärztliche Verordnung gegen das Originalpräparat ausgetauscht werden. Bisher war es so festgelegt, dass die Ärzte die Indikation für einen Austausch stellen. Im Januar 2020 äußerte sich der Vizepräsident der deutschen Rheuma-Liga, Dieter Wiek, im *Deutschen Ärzteblatt* dazu (HK/aerzteblatt.de, 2020). Zusammengefasst stellte er fest, „dass es ohne ein intensives Patientengespräch von Patientenseite zu einem Abbrechen der Therapie kommen könnte, da sich ein Nocebo-Effekt bilden kann, oder neu auftretende Nebenwirkungen schwer nachzuvollziehen sind. Hierbei sollte auch unterschieden werden, ob Patienten bereits mit einem Biosimilar behandelt wurden und auf ein anderes Biosimilar wechseln, oder ob der Wechsel von Originalpräparat zu Biosimilar stattfindet.“ [12]

Biosimilars dürfen nicht ohne ärztliche Verordnung gegen das Originalpräparat ausgetauscht werden

In einigen Austauschstudien zeigten sich diesbezüglich bei der rheumatoiden Arthritis keine signifikanten Unterschiede im Hinblick auf Wirkung und Nebenwirkung [13]. In der offenen PLANETRA-Extensions-Studie wurden 302 RA-Patienten eingeschlossen, wobei die Hälfte der Patienten das Biosimilar Infliximab (Inflectra, Remsima) intravenös über 102 Wochen erhielt und die andere Hälfte der Patienten erst mit dem Referenzarzneimittel Infliximab (Remicade) begann und dann nach 52 Wochen auf das Biosimilar wechselte. Auch hier fanden sich keine signifikanten Unterschiede zwischen dem ACR20/50/70-Ansprechen sowie dem Nachweis von spezifischen Anti-Drug-Antikörpern. Auch im Hinblick auf die Nebenwirkungen und Sicherheit verhielten sich die beiden Substanzen ähnlich. Die häufigsten Nebenwirkungen waren Reaktionen auf die Infusion, erhöhte Leberwerte und respiratorische Infekte [14].

In einer weiteren Studie mit Infliximab konnte nachgewiesen werden, dass ein Wechsel vom Referenzarzneimittel Infliximab zu einem Biosimilar dazu führte, dass ein erneuter Wechsel des Arzneimittels häufiger erfolgte. So wechselten die Patienten, die initial auf ein Biosimilar umgestellt wurden, 2‑ bis 3‑mal häufiger auf ein anderes Biologikum als Patienten, die nicht vom Referenzarzneimittel auf ein Biosimilar umgestellt wurden. Überwiegend wurden die Patienten (200 von 249 Patienten; insgesamt 80,3 %) auf das ursprüngliche Referenzarzneimittel umgestellt. Darüber hinaus zeigte sich, dass die Patienten, die vom Referenzarzneimittel auf das Biosimilar wechselten, 1,25-fach häufiger ihre Therapie unterbrachen als die Patienten, die beim Referenzarzneimittel blieben. Gründe für diese Wechsel konnte die Studie allerdings nicht nennen [15].

Hinsichtlich Rituximab wurde in einer Vergleichsstudie bei rheumatoider Arthritis ein Wechsel vom Referenzarzneimittel Rituximab auf ein Biosimilar evaluiert. Alle Patienten bekamen zunächst das Referenzarzneimittel. Anschließend wechselten alle Studienteilnehmer auf das Biosimilar, wenn sowohl der behandelnde Arzt als auch der Patient einverstanden waren. Daraus ergaben sich 2 Gruppen: die Gruppe, in der gewechselt wurde (255 Patienten), und die, die nicht wechselten (82 Patienten). Es konnte gezeigt werden, dass im Follow-up über 4 Monate keine signifikante Differenz im DAS28-CRP-Score zwischen den Gruppen bestand. Jedoch zeigten sich in der Biosimilargruppe ein Rückwechsel auf das Referenzarzneimittel aufgrund eines Wirkverlustes bei 16,5 % der Patienten sowie ein Wechsel aufgrund des Auftretens unerwünschter Arzneimittelwirkungen von 2,0 %. Risikofaktoren für einen solchen Wechsel waren Komorbiditäten sowie mindestens 2 biologische „disease-modifying antirheumatic drugs“ (bsDMARD) in der Anamnese. Im Verlauf wechselten 72,3 % der Patienten vom Biosimilar wieder zurück auf das Referenzarzneimittel, während 27,7 % auf ein anderes bsDMARD wechselten; 93,3 % der Patienten, die einen Rückwechsel aufgrund eines Wirkverlustes vollzogen, blieben über einen Nachbeobachtungszeitraum von fast 8 Monaten beim Referenzarzneimittel. Die Autoren schlussfolgern, dass ein Wechsel effektiv sein kann, die Risikofaktoren für einen Rückwechsel allerdings beachtet werden sollten [16].

In einer anderen doppelverblindeten, randomisierten Studie mit RA-Patienten wurde das Biologikum Etanercept über 52 Wochen mit dem Biosimilar Benepali oder Brenzys verglichen. Bei guter Verträglichkeit wurden die Patienten in einer offenen Studie über weitere 48 Wochen kontrolliert; 245 Patienten nahmen insgesamt teil, wobei auch hier die Hälfte von Beginn an mit Biosimilar behandelt wurde und die andere Hälfte der Patienten erst nach 52 Wochen auf Biosimilars umgestellt wurde. Außer einem gleichwertigen ACR-Ansprechen zwischen der Woche 52 und 100 zeigte sich ein ähnlicher Verlauf im radiologischen Progress. Spezifische Antikörper gegen Biologika entwickelten sich nur bei jeweils einem Patienten in beiden Gruppen und hatten keinen Einfluss auf die Wirkung. Die Sicherheit der beiden Substanzen nach 52 Wochen war vergleichbar, obwohl die Studie nicht darauf ausgelegt war. Erfreulicherweise fanden sich in beiden Gruppen keine Spritzreaktionen oder Auftreten einer aktiven Tuberkulose [10].

Kay et al. wies darauf hin, dass bei einem Austausch von einem Referenzarzneimittel auf ein Biosimilar darauf geachtet werden sollte, dass die gleichen Ergebnisse wie beim Referenzarzneimittel erzielt werden. Ein Wechsel scheint nur dann sinnvoll, wenn sich dadurch auch eine Kostenersparnis zeigt. Sollten Patienten nicht auf ein Referenzarzneimittel angesprochen haben, ist ein Wechsel auf das Biosimilar nicht zu empfehlen. Hier sollte v. a. erst gewechselt werden, wenn eine stabile Krankheitsaktivität besteht. Ein ständiger Wechsel von Biosimilar zum Referenzarzneimittel und umgekehrt sollte vermieden werden [13].

## Patientenzustimmung beim Wechsel auf Biosimilar

Interessanterweise scheint v. a. das ärztliche Gespräch vor einem Austausch auf ein Biosimilar eine große Bedeutung zu haben. In einer kontrollierten, randomisierten Studie aus Neuseeland wurden 96 Patienten mit einer rheumatologischen Erkrankung auf den Wechsel auf ein Biosimilar vorbereitet. Hier wurden die Patienten in 4 unterschiedliche Gruppen unterteilt. Eine Gruppe bekam ein Video mit einer positiven Erklärung zu Biosimilars gezeigt, die andere Gruppe zusätzlich zu dem Video noch einen positiven Vergleich, wie z. B. das Backen von Brot mit günstigerem Mehl. In dem Video äußerte sich der Arzt nicht nur verbal positiv gegenüber Biosimilars, sondern fiel auch durch eine positive Körpersprache auf. In der anderen Gruppe spielte sich dasselbe Szenarium mit einer negativen Erklärung zu Biosimilars bzw. negativem Vergleich ab. In der Auswertung zeigten sich signifikante Unterschiede in der Bereitschaft zum Wechsel und in der Effektivität von Biosimilars. Unabhängig von der Gruppeneinteilung wünschen sich Patienten vor einem Wechsel ein ausführliches Gespräch mit dem Arzt und Informationen zu Biosimilars zum Durchlesen [17].

Abschließend kann somit gesagt werden, dass ein Wechsel von Biologika auf Biosimilar möglich ist unter der Bedingung, dass die Indikation von einem Arzt gestellt wird und im Voraus ein ausführliches Gespräch mit dem Patienten erfolgt ist.

## Regionale Unterschiede im Einsatz von Biosimilars

Die Verordnung von Biosimilars sollte prinzipiell eine ärztliche Aufgabe sein. Oft wird die Verordnung jedoch von den kassenärztlichen Vereinigungen vorgegeben bzw. empfohlen. Wortwörtlich steht im Auszug aus der Vereinbarung mit den Krankenkassen „Kassenärztliche Bundesvereinigung und GKV-Spitzenverband, Rahmenvorgaben Arzneimittel 2020“: „Mit regionalen Zielvereinbarungen sollen die Vertragsärzte angeleitet werden, durch Verlagerung der Verordnungen hin zur Leitsubstanz und zu rabattierten bzw. preisgünstigen Arzneimitteln sowie zu wirtschaftlichen Versorgungsalternativen noch vorhandene Wirtschaftlichkeitsreserven zu erschließen … Innerhalb der Quoten sollen bevorzugt generische und rabattierte Arzneimittel verordnet werden. Die Nutzung von Biosimilars und Generika soll gefördert werden.“ Weiterhin wird auch hier regional unterschieden: „Die regionalen Vertragspartner können vereinbaren, dass bei der Bewertung der Zielerreichung Verordnungen vergleichsweise günstiger Substanzen oder rabattierter Arzneimittel berücksichtigt werden. Außerdem sind gegebenenfalls weitere auf der Landesebene vereinbarte Leitsubstanzen und Arzneimittelgruppen/Leitsubstanz(en) zu berücksichtigen.“ [18]

So zeigen sich deutliche regionale Unterschiede in dem Einsatz von Biosimilars (Tab. 2).

**Table 2:** 

Arzneimittelgruppe (Verordnungsmindestquote)	C03 Etanercept (in %)	C05 Rituximab (in %)	C02 Infliximab (in %)	C10 Adalimumab (in %)
KV	(„Biosimilares“ Etanercept)	(„Biosimilares“ Rituximab)	(„Biosimilares“ Infliximab)	(„Biosimilares“ Adalimumab)
Baden-Württemberg	53,7	77,0	45,5	30,1
Bayern	70,8	86,4	65,5	36,1
Berlin	42,5	64,7	36,8	22,4
Brandenburg	41,2	57,2	46,4	26,6
Bremen	56,8	66,2	70,0	37,0
Hamburg	68,4	71,5	67,7	38,4
Hessen	55,0	74,5	61,8	29,4
Mecklenburg-Vorpommern	45,6	55,1	52,0	24,9
Niedersachsen	75,9	83,1	84,6	53,9
Nordrhein	72,9	86,1	66,1	42,6
Rheinland-Pfalz	67,7	75,0	65,8	39,4
Saarland	50,9	58,5	51,1	25,5
Sachsen	49,4	79,2	46,1	27,3
Sachsen-Anhalt	51,8	73,2	42,7	26,3
Schleswig-Holstein	74,9	94,2	88,1	53,6
Thüringen	43,2	87,6	59,5	25,5
Westfalen-Lippe	80,0	91,9	82,8	49,9

*KV* Kassenärztliche Vereinigung

## Ökonomischer Nutzen/Kosteneinsparung durch Biosimilars

Laut des wissenschaftlichen Instituts der AOK konnten 2019 durch Umstellung von Referenzarzneimittel auf Biosimilar 459 Mio. € in Deutschland eingespart werden. Wäre aber systematisch das günstigere Produkt verschrieben worden, wäre sogar eine Einsparung bis 791,6 Mio. € möglich gewesen [20]. Hierbei liegt das Problem aber nicht nur bei den verschreibenden Ärzten, sondern ist sicherlich komplexer. Diese kennen oft die tatsächlichen Kosten für z. B. rabattierte Originalpräparate nicht und werden diesbezüglich auch nicht von den Krankenkassen informiert. Ein weiterer Punkt, der in der Datenerhebung nicht berücksichtigt wurde, aber sicherlich ein entscheidender Faktor ist, ist die benötigte Zustimmung des Patienten, bevor auf ein Biosimilar umgestellt werden kann.

Etwa ein Fünftel der Einsparungen geht auf die Biosimilars von Adalimumab zurück. Auch wenn bereits 45,5 % der Biosimilars von Adalimumab auf dem Markt vertrieben werden, gibt es dennoch einen Preisunterschied von 37,0 % Nettokosten zwischen dem Originalpräparat und den im Jahr 2019 bekannten 5 Biosimilars. Allein durch die Umstellung von Referenzarzneimittel auf Biosimilar konnten 2019 bei Adalimumab 205,2 Mio. € in Deutschland eingespart werden [20].

Auch bei dem Biosimilar Infliximab waren Einsparungen von 14,0 Mio. € pro Jahr in Deutschland möglich. Knapp 103,6 Mio. € an Einsparungen blieben ungenutzt [20].

In einer Studie von Uhrmann et al. wurde ein gesundheitsökonomisches Modell für Patienten mit ankylosierender Spondylitis entwickelt, das die Kostenersparnis von Inflectra gegenüber Remicade aufzeigen sollte [21]. Interessanterweise wurden hier nicht nur die direkten Kosten, die durch Arzneimittel- und Behandlungskosten entstanden, berechnet, sondern auch die indirekten Kosten, die durch Erwerbsminderung und Arbeitsunfähigkeit entstehen. Bei 10.000 virtuellen Patienten, die erfasst wurden, fielen unter Inflectra über Lebenszeit 92.631,62 € direkte Kosten inklusive Mehrwertsteuer und Zwangsrabatt an, im Gegensatz stiegen die direkten Kosten bei Remicade auf 116.205,17 €.

Insgesamt spielen beim Einsatz von Biosimilars unterschiedliche gesetzliche Rahmenbedingungen eine Rolle, wie z. B. die Verordnungsquoten, die von der Kassenärztlichen Bundesvereinigung jährlich vorgegeben werden und bestimmen, wie viel mindestens oder höchstens von einer bestimmten Arzneimittelgruppe ausgegeben werden darf. Hierbei ist die Spanne zwischen den Verordnungsmindestquoten der 17KVen (Kassenärztliche Vereinigungen) entsprechend groß [20].

So zeigte sich in einer Untersuchung des Marktforschungsinstituts „Insight Health“ für die Arbeitsgemeinschaft Pro Biosimilars für das Jahr 2019 eine Einsparung von 328 Mio. €, wenn in allen 17 KV-Regionen Biosimilars so häufig verschrieben werden würden wie im Bereich der KV mit dem höchsten Biosimilaranteil. Zusammen mit den bereits eingesparten 343 Mio. € könnte dann eine zusätzliche Einsparung von insgesamt 671 Mio. € erzielt werden [22].

**Figure 1:**
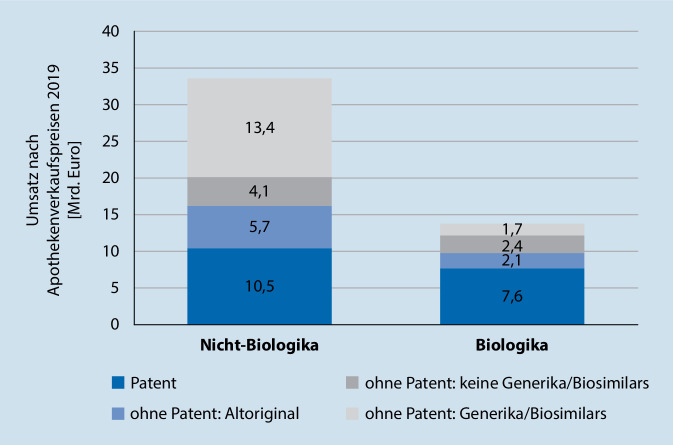

Weiterhin können bestimmte Arzneimittel einer Festbetragsgruppe zugeordnet werden mit der eine Erstattungshöchstgrenze einhergeht. Bei Biosimilars wird dieses Prinzip noch nicht ausreichend genutzt. Hier gibt es aktuell nur 5 Biosimilars, darunter Etanercept und Infliximab, die einer Festbetragsgruppe angehören (Stand 28.02.2020). Abschließend gibt es noch Rabattverträge, die direkt zwischen Krankenkasse und Hersteller abgeschlossen werden. Hierbei ist die Apotheke aber bisher nicht verpflichtet, das biosimilarfähige Arzneimittel abzugeben ([20]: Abb. 1).

Ein weiterer Faktor, der die Preislage von Biosimilars bestimmt, sind die Preissenkungen der Referenzarzneimittel.

Für TNF-α-Inhibitoren wurde gezeigt, dass die Preissenkung des Referenzarzneimittels den Marktanteil des Biosimilars bestimmt. Eventuell könnte eine zu starke Preissenkung zu einem verzögerten Markteintritt von Biosimilars führen bzw. diesen verhindern [23].

Interessanterweise beeinflussen Biosimilars nicht nur den Preis der Referenzarzneimittel, sondern auch die Preise für Biologika, für die es noch gar kein Biosimilar gibt. Das Ausmaß der Preissenkung der gesamten Medikamentengruppe kann dann fast der Preissenkung des Referenzarzneimittels entsprechen [23]. Mit Spannung werden daher neue Biosimilars erwartet, da 2020 bereits 18 Biologika aus unterschiedlichen Fachrichtungen ihren Patentschutz verloren haben [25].

## Fazit für die Praxis

- Insgesamt gibt es verschiedene Biosimilars zu den Biologika Adalimumab, Etanercept, Infliximab und dem B‑Zell-Antagonisten Rituximab, die bei der Therapie der RA (rheumatoide Arthritis) zum Einsatz kommen.
- Aussagekräftige Daten legen sowohl die Sicherheit als auch die Wirksamkeit von Biosimilars nahe. Insbesondere ermöglichen sie uns ein finanzielles Einsparungspotenzial, das gesundheitsökonomisch von großer Bedeutung ist. Die Verschreibungszahlen variieren dennoch regional, bisher sind die Gründe dafür noch unklar.
- Mit Spannung werden neue Biosimilars erwartet, da 2020 18 Biologika aus unterschiedlichen Fachbereichen ihren Patentschutz verlieren, darunter z. B. Tocilizumab, ein Interleukin-6-Rezeptor-Antagonist, zugelassen für die rheumatoide Arthritis.
